# Surface-Acoustic-Wave Sensor Design for Acceleration Measurement

**DOI:** 10.3390/s18072301

**Published:** 2018-07-16

**Authors:** Sergey Shevchenko, Alexander Kukaev, Maria Khivrich, Dmitry Lukyanov

**Affiliations:** Department of Laser Measurement and Navigation Systems, Saint Petersburg Electrotechnical University, Professora Popova 5, 197376 Saint Petersburg, Russia; askukaev@gmail.com (A.K.); mariya-khivrich@yandex.ru (M.K.); dplukyanov@mail.ru (D.L.)

**Keywords:** microaccelerometer, surface acoustic waves (SAWs), interdigital transducer (IDT), quartz packaging

## Abstract

We suggest a concept design of a SAW-based microaccelerometer with an original triangular-shaped console-type sensing element. Our design is particularly optimized to increase the robustness against positioning errors of the SAW resonators on the opposite sides of the console. We also describe the results of computer simulations and laboratory tests that are in a perfect agreement with each other and present the sensitivity characteristics of a manufactured experimental design device.

## 1. Introduction

In recent years, micromechanical sensors found a variety of applications ranging from aerospace, vehicle and automotive industry to consumer goods such as personal navigation tools, smartphones, video cameras and many other devices with embedded micro-electromechanical systems (MEMS) [1]. Currently the most commonly utilized technology in the field is provided by the stress-sensitive semiconductor elements. Based on the physical principles that date back to the late 1950s–early 1960s, during the last half century due to the success of microelectronics this technology exhibited a drastic compaction of sensitive elements that finally led to broadening of its application area as well as to the drastic increase in production volume. In particular, typical microsensor size reduced more than 100-fold from several square centimeters to about one square millimeter during the last decade, while its production volume has increased more than two-fold. Several alternative principles have been suggested, such as electrostatic stiffness variation [2] and surface micromachining [3]. The major fabrication technologies include photolithography and etching to produce the required shapes [4]. While the majority of MEMS sensors are nowadays silicon based, also metal-based and ceramic-based sensors [5] have been developed, finding mainly special applications such as medical instrumentation and implantable sensors, due to their biocompatibility.

One of the key applications of micromechanical sensors are inertial integrated navigation systems that currently find an increasing number of applications in various mobile vehicles. Recent progress in the microelectronic technology provides the opportunity to design and to manufacture such devices as solid-state accelerometers (SSAs) that are currently of urgent demand for multiple civilian and military applications, such as biomedical wearable and implantable devices [6], industrial robots [7], automobiles [8], aircrafts, drones [9], satellites [10] as well as personal navigation equipment [11].

However, the majority of commercially available silicon microaccelerometers (SMAs) are not solid-state in the true sense. Generally, they utilize discrete elastic suspensions that provide the necessary degrees of freedom for inertial masses that, in turn, require that the microoscillating system is installed with a high precision of 0.1–1 μm. These conditions reduce both the vibrostability and the shock resistance of SMAs while requiring high-tech manufacturing technology [12].

In contrast, a specific class of devices and systems that employ the surface acoustic wave (SAW) properties in piezoelectric crystals is largely free of these limitations and thus its utilization in perspective microaccelerometer design is largely promising [13,14,15,16,17]. In this paper, we consider a concept design of a SAW-based microaccelerometer. Its potential advantages over conventional micromechanical analogs include:

- simplicity of the sensing element kinematic scheme;
- high-level design integrity (i.e., fewer component parts and joints used);
- lack of extra discrete elastic suspensions;
- minimal use of conventional planar microelectronic technologies, thus minimizing the SSA production cost.

## 2. Results

### 2.1. SAW Microaccelerometer Design Concept

**Figure 1:**
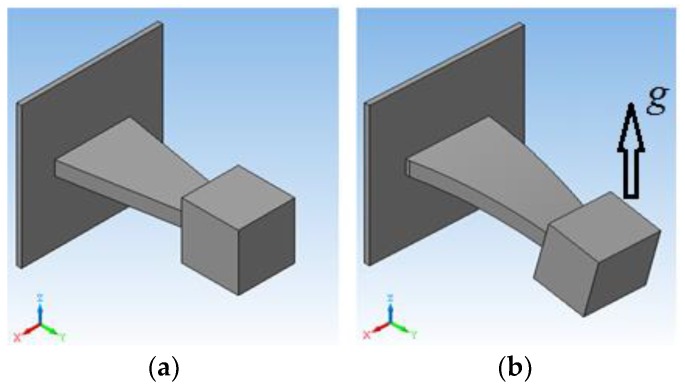
The overall sensitive element design shape (**a**) without external acceleration applied; (**b**) under external acceleration applied.

The conceptual SAW microaccelerometer design incorporates two main parts, namely a micromechanical sensitive element (SE) and an electronic signal-converting circuit. The experience in the development of SAW transducers [18,19] indicates that the console construction of an SE loaded with a proof mass offers the highest sensitivity (Figure 1).

Figure 1 depicts the overall triangular-shaped sensitive element design. The piezoelectric sensitive console is fixed at one end at the case wall while being loaded by inertial mass at the other end. Once external acceleration *g* is applied, the console exhibits elastic deformation that is proportional to the applied acceleration.

**Figure 2:**
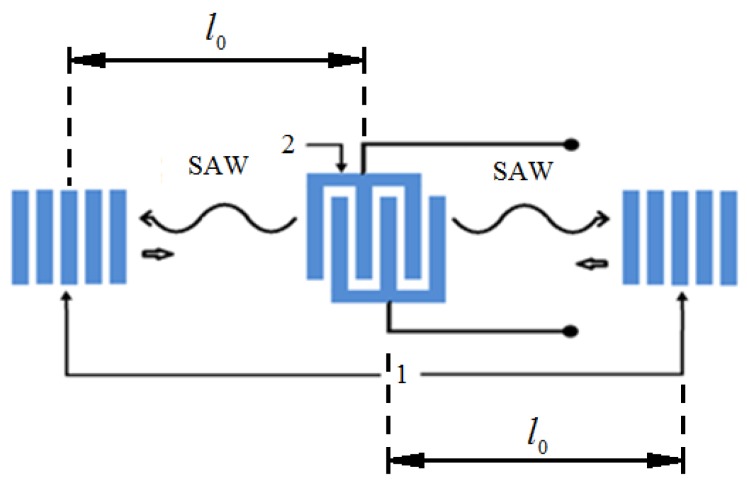
Surface acoustic wave resonator schematic illustration. 1–reflectors; 2—interdigital transducer (IDT).

Figure 2 illustrates the operational principles of the SAW resonator.

**Figure 3:**
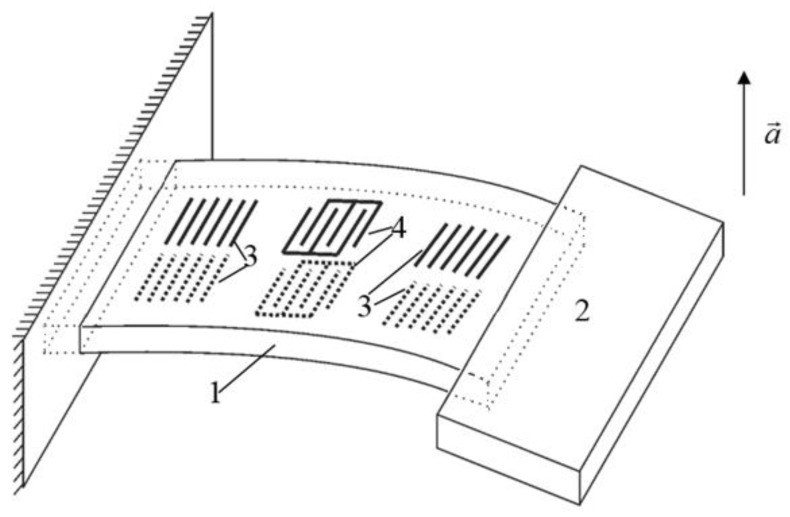
The overall schematic design of the SAW-based accelerometer.

The IDT 2 induces two reciprocally directed running acoustic waves that are next reflected from reflectors 1 thus forming a standing wave. On each side of the sensitive console, using the method of photolithography (which is widely used for the production of SAW devices), the structure of the SAW resonators is produced (see Figure 1). The console can be made directly from a material with piezoelectric properties. This simplifies the manufacturing technology of the accelerometer and allows direct formation of the resonator directly on the surface of the piezoelectric without the use of intermediate layers. Therefore, as the material of the console, a crystalline quartz of the ST cut was selected, which has a high thermal stability. After that, one end of the sensitive console 1 is fixed to the base by means of an adhesive joint, and its other end is loaded with inertial mass 2. When external acceleration is applied and the console bends, the distance travelled by the SAW between the IDT and the resonator changes proportionally to the applied acceleration (see also Figure 3).

On the opposite sides of the console, SAW resonators are located, formed by the IDT 3 and reflectors 4. When subjected to acceleration, the beam undergoes bending deformations and, as a result, stretches and contracts the opposite surfaces of the console. The resulting surface stresses change the elastic modulus and the density of the material in the surface layer, as well as its geometric dimensions. During stretching deformations, the SAW propagation time along the surface of the console increases both as a result of its geometric elongation and due to the decrease in its phase velocity, the variations of which are determined by the change in the elastic modulus. On the opposite side of the plate, compression deformations occur, leading to the opposite change in the SAW propagation time.

The characteristic frequencies of the resonators are determined by the distance between the reflectors, their dispersion properties, and the SAW propagation velocity. The characteristic frequency of the unperturbed resonator $f_{0}$ can be found from:

(1) $$f_{0}=u_{0}q/2l_{0},$$

where $u_{0}$ is the unperturbed SAW phase velocity, q>>1 is an integer constant that depends on the console material, and $l_{0}$ is the length between effective centers of the reflector and IDT (Figure 2) thus yielding:

(2) $$f_{0}=\frac{q}{2\tau_{0}},$$

where $\tau_{0}$ is the SAW transmission time over distance $l_{0}$.

**Figure 4:**
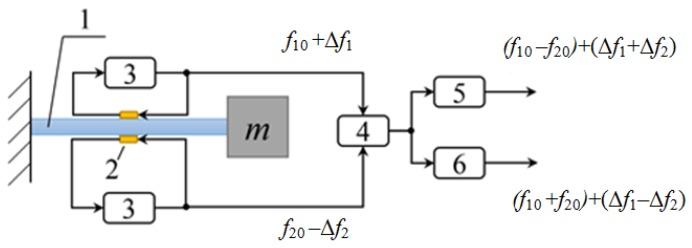
Operational scheme of the SAW accelerometer. Figure adapted from [12].

Then, the characteristic frequencies of the resonators $\left(f_{1},f_{2}\right)$ and their differences can be determined as:

(3) $$\begin{array}{l} f_{1,2}=\frac{q}{2\tau_{0}(1\pm \mu )}=f_{0}(1\mp \mu )\\ f_{2}-f_{1}=2f_{0}\mu \end{array},$$

where μ is the average SAW travel-time deviation along the resonator under external mechanical stresses. Here, a negative sign denotes stretching, while a positive sign denotes compression. As a result, a differential SAW accelerometer design can be suggested based on a simple scheme depicted in Figure 4.

The overall operational scheme of the SAW accelerometer is shown in Figure 4. The bending of the sensitive console 1 affects the characteristic frequencies of the SAW resonators 2 powered by amplifiers 3 and combined at the mixer 4 that in turn are registered by changing the combination frequencies:

*F_dif_* = (*f*_10_ − *f*_20_) + 2 Δ*f,* (4)

*F*_sum_ = (*f*_10_ + *f*_20_). (5)

### 2.2. SSA Console Shape Optimization

**Figure 5:**
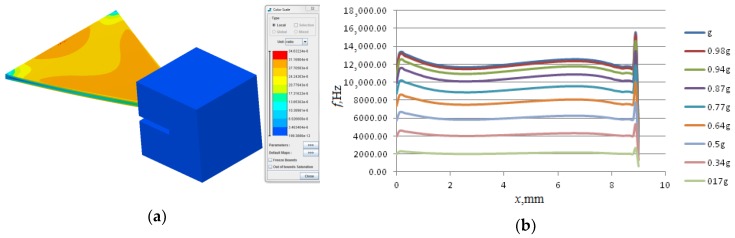
Surface acoustic wave solid-state accelerometer (SSA) with a triangular-shaped sensitive element (SE): (**a**)—simulation results of the linear acceleration influence on the characteristics of SE of SSA on SAW with triangular console shape; (**b**)—the distribution of relative deformations along the central longitudinal axis.

Next we designed a 3D model of the described SE using OOFELIE: Multiphysics. Isotropic quartz was chosen as the console material, while W/Ni/Mo alloy was chosen as the inertial mass material. External acceleration application was defined as uniformly distributed gravity acceleration that was directed along the *y*-axis. One end of the console was clamped firmly. The simulation results of the influence of the applied linear acceleration on the characteristics of SE of SSA on SAW with triangular shape of console are shown in Figure 5.

In order to maintain equal distributions of normal stresses on any cross-section of the console, a variable console width *b*(*x*) is chosen such that the SAW propagation velocity is constant along the console. Triangular-shaped console design is a solution that provides the equal bending resistance. The equation that relates the variable width of the console with the longitudinal coordinate and relevant expressions for internal stresses σ(*x*) as well as relative deformations ε(*x*) is given by [12].

(6) $$b(x)=b(l_{1})\cdot \left(1+2\frac{l_{1}-x}{l_{2}}\right)$$

(7) $$\sigma (x)=\frac{6m_{2}a(l_{1}-x)}{b(x)h_{1}^{2}}$$

(8) $$\varepsilon (x)=\frac{6m_{2}a(l_{1}-x)}{b(x)h_{1}^{2}E}$$

Assuming *m*_2_ >> *m*_1_ leads to the ansatz *m*_1_ = 0. According to Equation (6), in order to provide the uniform distribution of the relative deformations along the console, its side faces should converge in the center of a loading mass.

The simulation results of a linear acceleration influence on the characteristics of the SAW SSA with a triangular-shaped SE console are given in Figure 5.

Figure 5 shows that the triangular-shaped console provides a significant increase of the uniformity of the distribution of the relative deformations on its opposite surfaces indicated by a prolonged plateau on the relative deformations plot (see Figure 5b) that is sufficient to accommodate a SAW resonator taking into account the available manufacturing accuracy [12,20].

**Figure 6:**
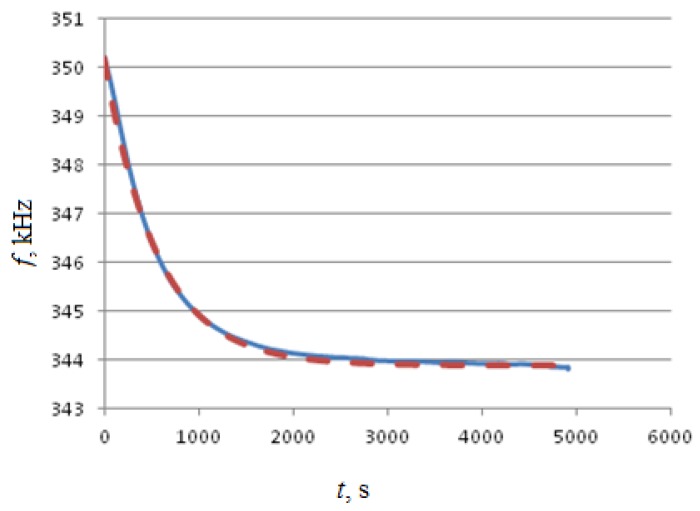
Initial transition process at startup of the experimental design. Full line shows experimental data, dashed line shows simulation data.

Next, a series of experimental tests have been performed with a prefabricated console element to confirm its characteristics predicted previously by computer simulations only (Figure 6).

Figure 6 shows averaged results of three experimental tests for the same prototype device.

**Figure 7:**
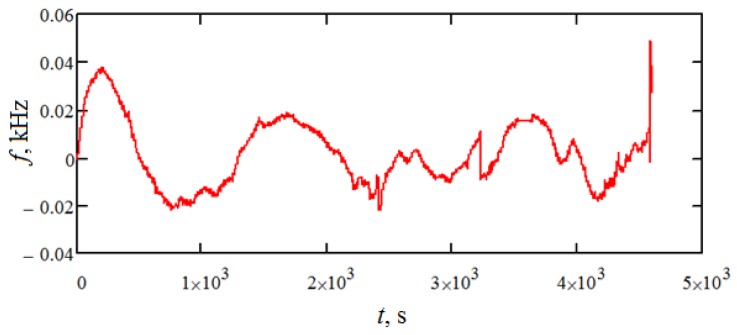
The noise component of the sample output signal.

After subtracting the obtained function from the experimental data, the noise component has the form shown in Figure 7.

**Figure 8:**
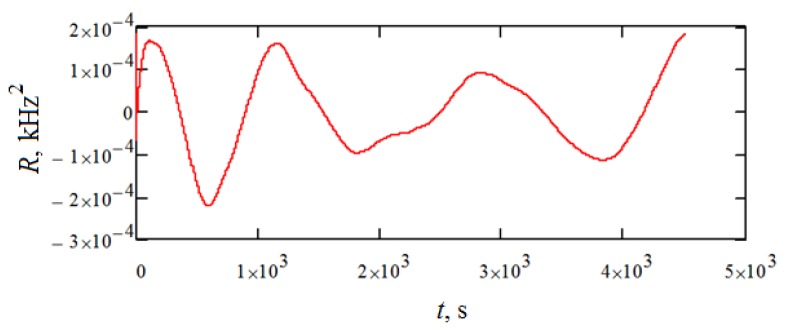
Correlation function.

**Figure 9:**
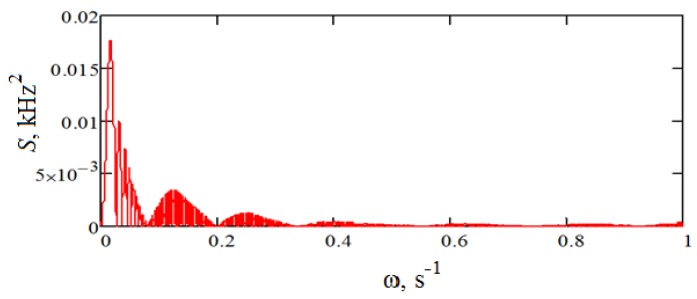
Power spectral density.

The characteristics of the noise obtained are presented in Table 1. Also, the correlation function and the spectral density of the resulting noise are shown in Figure 8 and Figure 9, respectively.

**Figure 10:**
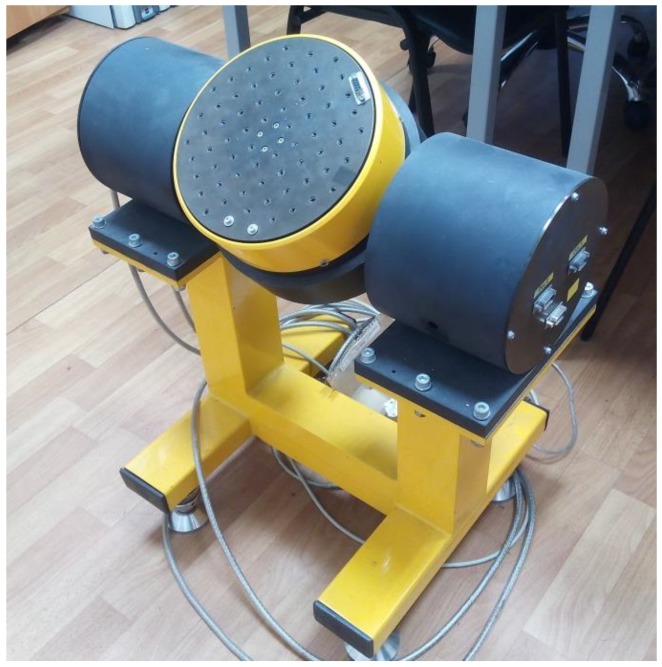
Two-axis rate-table design.

Next, the calibration of the experimental design with the triangular shape of the sensitive element was performed in the gravitational field of the Earth and its main characteristics were investigated. The graduation was carried out on an original two-axis rate-table design (ZG Optique SA, St-Aubin, Switzerland) described in detail [21] as depicted in Figure 10. The studies were carried out at room temperature (25 ± 5 °C) between 40% and 60% relative humidity. The above conditions were chosen due to material temperature frequency characteristics that exhibit a maximum around 25 °C, while at other temperatures additional corrections are required. Since similar studies of the same material have been carried out previously by multiple groups, we did not carry them out any more (for a consensus review, we refer to Figure 5.15 in [22]).

**Figure 11:**
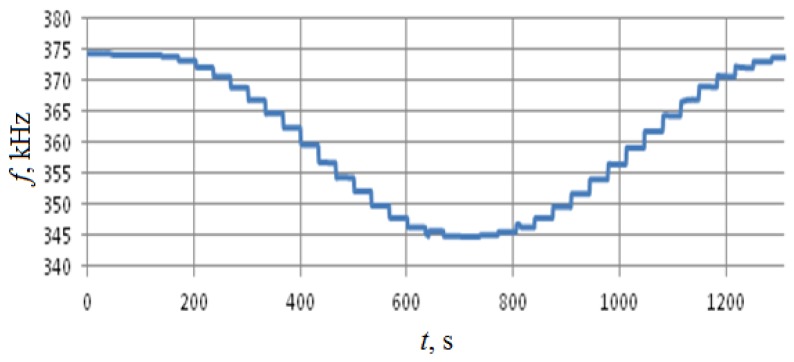
Test results for rotations around the *z*-axis.

**Figure 12:**
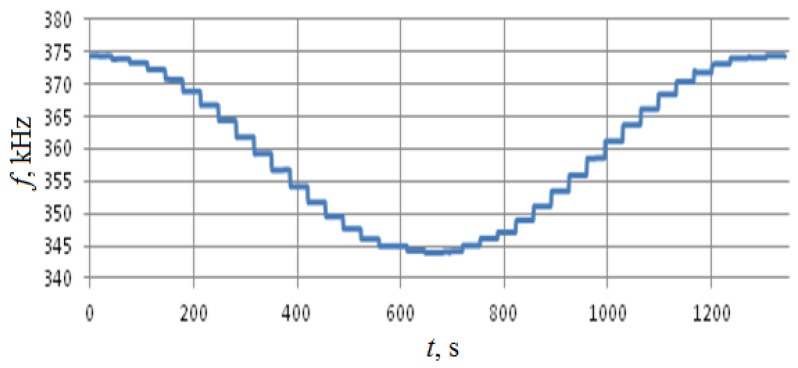
Test results for rotations around the *x*-axis.

**Figure 13:**
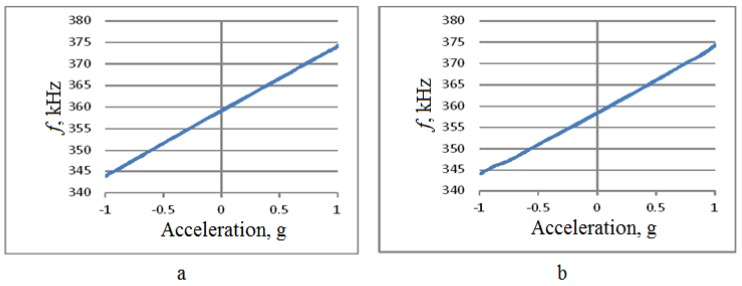
The output characteristic for rotations around the *z*-axis. (**а**)—Rotation from −180° to 0°; (**b**)—rotation from 0° to +180°.

**Figure 14:**
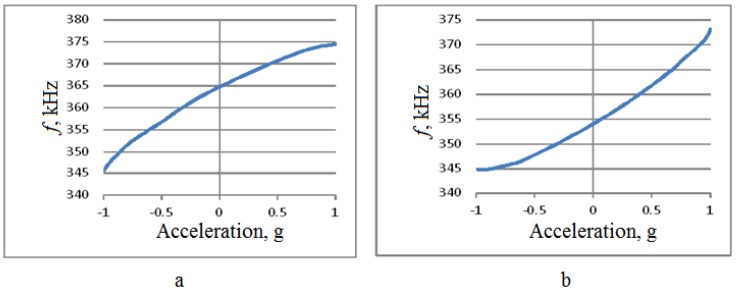
The output characteristic for rotations around the *x*-axis. (**а**)—rotation from −180° to 0°, (**b**)—rotation from 0° to +180°.

Calibration was performed along the two horizontal axes, so the rotation was performed around axes x and z with 10° angular resolution, see Figure 3. Test results are summarized in Figure 11, Figure 12, Figure 13 and Figure 14.

Finally, the output characteristic for the same axes was obtained. The acceleration was first increased from –g to g and next was reduced back to –g, thus testing the reproducibility of the sensitive element’s reaction to external acceleration.

As shown in Figure 11 and Figure 12, the change in the output signal during rotation around the z- and x-axes is almost the same and is about 30 kHz. However, when rotating around the *x*-axis, that is, measuring the acceleration along the *z*-axis, back-and-forth acceleration change induces a hysteresis-shaped effect in the sensitivity characteristics. Thus the reproducibility of the results for *x*- and *z*-axes is significantly different (Figure 13 and Figure 14). Besides this notable effect, the design is generally multi-axial. The obtained characteristics are summarized in Table 2.

**Figure 15:**
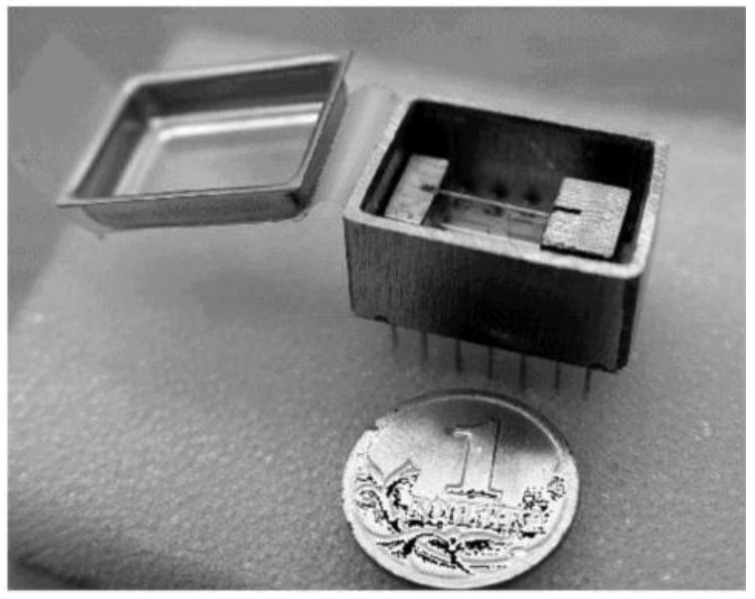
Experimental accelerometer design. Coin with approximately 1-cm diameter is shown for comparison.

**Figure 16:**
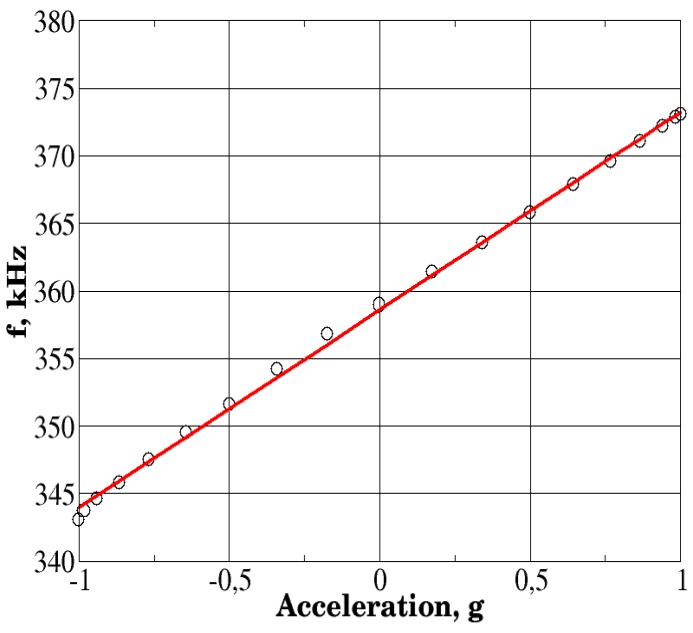
Dependence of the output frequency of the device on the applied acceleration.

These results were next compared against the results of laboratory tests of an accelerometer prototype (Figure 15). The accelerometer output characteristic obtained in laboratory tests is shown in Figure 16.

Experimental data that are shown in Figure 16 (by circles) have been averaged over a series of three independent measurements that were performed on the same prototype device. This averaging, while reducing random instrumental measurement error, did not affect any potential systematic error caused by, for example, sensitive element asymmetry. The result has been compared with the linear model obtained by the least-squares fit of the averaged experimental data. The results were next used to obtain the nonlinearity characteristics of the fabricated prototype device.

Direct comparison of the simulation results and laboratory tests provides a perfect match. The sensitivity of the accelerometer is about 14.62 kHz/g, as obtained by both lab tests and computer simulations, while the relative standard deviation between the experimental data and the computer simulation results did not exceed 3% for the entire acceleration range shown. A nearly perfect match of the results indicates that even rather simplified models that do not take into account material anisotropy and piezoelectric effects may provide a very good characterization of the modeled finite element.

## 3. Conclusions

SAW-based accelerometers possess a number of significant advantages, such as high resistance to vibration and shock, low cost and simplicity of production. The suggested SAW SSA demonstrates improved characteristics due to the optimization of the SSA console shape design that is more robust against technological SAW-resonator disposition errors compared to the rectangular shape design. Obtained results were compared with the experimental data and demonstrated a perfect match that indicates high accuracy of the applied simulation technique. Both experimental and simulated data show that the sensitivity of an accelerometer is close to 15 kHz/g, which is approximately one-half of the sensitivity of a similar design based on a rectangular console [12,17]. Taking into account high sensitivity along with extremely high robustness, low-cost production and small sizes, we can conclude that SAW-based accelerometers are able to compete in the market with modern MEMS accelerometers.

The keynote advantage of the SAW-based accelerometers is the absence of any moving parts in their design, which could potentially make them extremely shock, vibration and temperature resistant. Recent literature data based solely on the results of computer simulations predict potential values up to several-dozen g [23,24], while no experimental confirmation has been provided. Although rather simple physical models describing the SAW accelerometer dynamics do not limit their shock or vibrational resistance, we understand that most likely the limiting factors would come from secondary effects such as fabrication precision, potential sensor element asymmetry, inhomogeneity of raw materials and so on. Accordingly, we believe that it is too early to provide any reasonable estimate of shock and vibration resistance prior to experimental tests, which are still pending at the moment.

## Figures and Tables

**Table 1:** Noise characteristics.

**Sample Mean, kHz**	1.425 × 10^−3^
**Sample Variance, (kHz)^2^**	1.823 × 10^−4^

**Table 2:** The keynote characteristics of the experimental design.

Characteristics	Rotations around the *z*-axis	Rotations around the *x*-axis
***k*_0_, kHz**	358.785	359.408
***k*_m_, kHz** /g	15.23	14.25
**Nonlinearity, %**	0.25	2.5
